# Efficient Screening of Combinatorial Peptide Libraries by Spatially Ordered Beads Immobilized on Conventional Glass Slides

**DOI:** 10.3390/ht8020011

**Published:** 2019-04-30

**Authors:** Timm Schwaar, Maike Lettow, Dario Remmler, Hans G. Börner, Michael G. Weller

**Affiliations:** 1 Division 1.5 Protein Analysis, Federal Institute for Materials Research and Testing (BAM), Richard-Willstätter-Strasse 11, 12489 Berlin, Germany; timm.schwaar@bam.de (T.S.); maike.lettow@fu-berlin.de (M.L.); dario.remmler@chemie.hu-berlin.de (D.R.); 2 Department of Chemistry, Humboldt-Universität zu Berlin, Brook-Taylor-Straße 2, 12489 Berlin, Germany; boernerh@hu-berlin.de; 3 Institut für Chemie und Biochemie der Freien Universität Berlin, Takustraße 3, 14195 Berlin, Germany

**Keywords:** peptide library, screening, HTS, biomarker, on-bead sequencing, on-chip sequencing, microarray, grid, bead array, drug screening, lead compounds, pharmaceutical development, affinity reagents

## Abstract

Screening of one-bead-one-compound (OBOC) libraries is a proven procedure for the identification of protein-binding ligands. The demand for binders with high affinity and specificity towards various targets has surged in the biomedical and pharmaceutical field in recent years. The traditional peptide screening involves tedious steps such as affinity selection, bead picking, sequencing, and characterization. Herein, we present a high-throughput “*all-on-one chip*” system to avoid slow and technically complex bead picking steps. On a traditional glass slide provided with an electrically conductive tape, beads of a combinatorial peptide library are aligned and immobilized by application of a precision sieve. Subsequently, the chip is incubated with a fluorophore-labeled target protein. In a fluorescence scan followed by matrix-assisted laser desorption/ionization (MALDI)-time of flight (TOF) mass spectrometry, high-affinity binders are directly and unambiguously sequenced with high accuracy without picking of the positive beads. The use of an optimized ladder sequencing approach improved the accuracy of the de-novo sequencing step to nearly 100%. The new technique was validated by employing a FLAG-based model system, identifying new peptide binders for the monoclonal M2 anti-FLAG antibody, and was finally utilized to search for IgG-binding peptides. In the present format, more than 30,000 beads can be screened on one slide.

## 1. Introduction

Peptides represent a class of pharmaceutical compounds, molecularly poised between small molecules and proteins, yet biochemically and therapeutically distinct from both [1]. Among various chemical library formats, one-bead-one-compound (OBOC) libraries are a proven approach for the identification of protein-binding ligands [2]. By applying the split-and-mix method, introduced by Lam et al., millions of compounds can be readily synthesized in parallel [3]. When these libraries are created on resins that have a hydrophilic surface to suppress nonspecific reagent binding, they can be used directly in binding screenings for the identification of ligands for proteins or other targets [4].

However, its potential has not yet been fully explored because of several technical obstacles, including the screening of millions of beads by examination under a fluorescence microscope and manually isolating the positive beads by hand picking. Finally, the peptide will be released from the bead and, subsequently, its sequence is determined, either directly by mass spectroscopic or indirectly by encoded beads.

### 1.1. High-Throughput Screening Techniques for OBOC Libraries

To avoid these complex and laborious steps, several improvements of the OBOC technology have been presented. A strategy for analyzing large libraries is bulk separation by protein-coated magnetic particles [5]. Alternatively, the separation can be done by sorting beads using a system designated as complex object parametric analyzer and sorter (COPAS), a modified flow cytometer (FACS), which is compatible with beads typically used in OBOC libraries with a diameter in the range of 70–90 µm [6]. Conventional FACS systems may also be used for the same purpose, keeping in mind that these devices are designed for analyzing cells in the size up to 10 µm [7]. For the automated isolation of positive hits from large OBOC libraries, Hintersteiner et al. published a sophisticated confocal nanoscanning and bead picking method (CONA), followed by sequencing of the binding peptides separately on a µHPLC/mass spectrometry (MS) instrument [8]. Wang et al. developed chip-based techniques to combine affinity experiments and the MS decoding of the peptide sequence. In a lithographic process, a microstructured microwell array was manufactured, entrapping a single bead in each silicon well. A capacity of 800 beads per chip was accomplished. On this silicon plate, fluorescence screening and sequencing by matrix-assisted laser desorption/ionization (MALDI)-MS/MS were combined [9].

Other approaches to screen combinatorial libraries comprise the use of phage display [10] and similar biological methods. Their library size can be very large. However, phage display libraries are largely limited to proteinogenic amino acids and show some other unpleasant properties, such as severe biases by parasitic sequences and other diversity deficiencies [11]. Recently, DNA-encoded libraries seem to have gained some popularity, particularly in industry. First proposed by Brenner & Lerner in 1992, the emergence of next-generation sequencing technology may have fostered their renaissance [12]. Their main application seems to be in the screening of small molecule libraries. The application to peptide libraries shows some disadvantages, such as “poor step economy relative to information yield” [13]. Therefore, we believe that the OBOC approach still shows great promise [14] for the screening of peptide libraries because of its extreme chemical flexibility; experimental simplicity; and by the application of advanced analytical techniques, which gained significantly improved performance during the last decades [15].

### 1.2. De Novo Peptide Sequencing from a Single Bead

The screening of OBOC libraries usually results in some positive beads, carrying multiple copies of the respective target-binding peptide. Each of these beads needs individual and accurate sequence determination. In the case of peptide libraries with a free N-terminus, Edman microsequencing can be used [3,16]. However, this approach is expensive and time-consuming, which greatly restricts its widespread application in high-throughput sequencing of OBOC libraries.

Today, mass spectrometry is the method of choice when it comes to the sequencing of multiple peptides in a short time. Particularly, tandem mass spectrometry (MS/MS) is well established in proteomics. MS/MS spectra, usually of chromatographically separated peptides produced by tryptic digestion of a protein, are translated into peptide sequences, normally supported by various databases including genetic information [17]. De novo sequencing of peptides by MS/MS from a single bead, on the other hand, is an even more challenging task. In the case of the sequencing of peptides from synthetic combinatorial libraries, database comparisons are not possible and the sequence identification completely relies on the quality of the fragmentation spectra [18]. In practice, the fragmentation processes in mass spectrometers are far from ideal and missing fragmentations can occur, making a sequence determination difficult and error-prone. Karas et al. noted in a paper that despite several advances in MS/MS in the past, a 100% sequence coverage may be “*a modern form of surrealism”* [19]. Furthermore, comparative studies of the accuracy of several de novo algorithms showed that usually only two-thirds of the residues in a peptide could be identified correctly [20,21].

Youngquist et al. introduced another encoding method, the so-called ladder synthesis approach for peptide library creation [22], which was later modified by St. Hilaire et al. [23]. Here, a small amount of a terminating reagent is added to each amino acid coupling step, generating the full-length products plus a small percentage of sequence-truncated products. For example, Fmoc- and Boc-protected amino acids, allowing deprotection of only the Fmoc-protected amines for continued synthesis and leaving truncated fragments after cleavage from the solid support [23]. Direct measurement of the peptide sequence is possible using matrix-assisted laser desorption/ionization (MALDI)-time of flight (TOF) MS in one step without any fragmentation. In comparison to the MS/MS approach, an easy, straightforward, and highly sensitive sequence read-out is possible, because of the deliberately generated ladder-like peaks in the spectra.

### 1.3. Linker For Liquid Free Peptide Cleavage 

The linker between polymer resin and peptide for a high-throughput analysis of peptide libraries should allow the liquid-free cleavage from the resin leaving the peptides on or in the respective bead. This is possible by using a photolabile linker [24], which is cleavable by UV-light prior to the sequencing by MALDI-TOF MS/MS [25]. It was shown that a direct on-bead-sequencing of peptides by MALDI-TOF MS/MS is possible [26]. Another approach is the release of resin-bound peptides by ammonia vapors using the 4-hydroxymethylbenzoic acid (HMBA)-linker [27]. So far, this linker has not been used for direct “on bead” sequencing but showed great promise in cleavage efficacy [28]. 

Here, we present a novel technique to identify protein binders from an OBOC library by immobilizing the beads on a modified glass slide, on which the beads have been aligned by a precision sieve. This also allows for a multiple step screening, in which the immobilized bead library is sequentially incubated with fluorophore-labeled reagents, identifying suitable peptides by a high-resolution fluorescence scan. In addition, a control screening (pre-screening) can be performed to weed out false-positives caused by beads with high autofluorescence or non-specific protein binding. To circumvent the need for a fragmentation step by MALDI-TOF MS/MS, which can result in incomplete sequence information, a simple encoding approach (*ladder sequencing*) has been adapted for this purpose. This allows for the peptide sequence identification by fragmentation-free MS with almost 100% accuracy.

## 2. Materials and Methods

### 2.1. Chip Preparation

The chip was prepared by attaching an electrically conductive double-sided adhesive tape to a glass microscope slide (75 × 25 mm^2^) and placing it in a custom-made holder. A metal sieve with a mesh of 100 µm is pressed on the surface of the chip and dried beads are spread on the sieve, which stick to the adhesive tape below. Finally, the sieve and any loosely bound beads are removed, resulting in a regular, grid- or array-like layer of beads, which is favorable for an overlapping-free MALDI-TOF MS sequencing process. 

### 2.2. On-Chip Protein Incubation

All incubation steps were performed directly on the chip surface in PBS-T BSA (pH 7.5; 0.1% Tween 20; 1% BSA) in a petri dish. Prior to the first incubation, the chip-bound beads were pre-swollen by covering the chip with PBS-T BSA and gentle shaking for one hour. The chip was washed five times with PBS-T BSA and incubated with the primary antibody (1 mg/mL; 1:10,000 diluted in PBS-T BSA) overnight, washed, and incubated with the same concentration of anti-mouse IgG-Atto633 antibody for one hour. The chip was washed and dried before performing high-resolution fluorescence imaging using a microarray scanner MArS (Ditabis AG) using a laser wavelength of 635 nm. For further experiments, all proteins were removed by treating the chip with 6 M guanidinium chloride solution and subsequent washing steps with high-purity lab water. 

### 2.3. On-Bead Peptide Ladder Sequencing by MALDI-TOF MS

The dried chip was placed in a chamber with concentrated ammonia solution at the bottom for two hours. The matrix application was achieved using a conventional airbrush gun. About 2 mL of the matrix solution was used per chip, consisting of 20 mg of 2,5-dihydroxyacetophenone in ethanol and diammonium hydrogen citrate (18 mg/mL in MilliQ lab water) (3:1 v/v). The matrix solution was applied from a distance of 13 cm and with a pressure of 2 bar. MALDI-TOF MS and MALDI-TOF MS/MS sequencing were performed using a Bruker autoflex II smartbeam™ mass spectrometer. Coordinates of positive peptides, obtained from the microarray scans, were transformed by linear regression and via calibration of four beads on the corners of the slide. Peptide mass spectra were directly acquired on the bead surface.

### 2.4. Resin preparation 

TentaGel HL NH_2_ beads (75 μm, 0.47 mmol/g) were swollen in dimethylformamide (DMF) for 1 h before usage. The linker was coupled on the resin by adding 4-(hydroxymethyl)benzoic acid (HMBA) (0.1 mol/L, 4 eq.) in DMF with O-(1H-6-chlorobenzotriazole-1-yl)-1,1,3,3-tetramethyluronium hexafluorophosphate (HCTU) (4 eq.), and N-methylmorpholine (NMM) (8 eq.) for 1 h. Glycine was incorporated at the C-terminus by adding Fmoc-Gly-OH (0.3 mol/L, 5 eq.) in dichloromethane (DCM) with diisopropyl carbodiimide (DIC) (2.5 eq.), followed by dropwise addition of a solution of dimethylaminopyridine (DMAP) dissolved in DMF (50 mmol/L, 0.1 eq.). The spacer sequence GGTERSG was synthesized by adding Fmoc-amino acids (0.1 mol/L, 4 eq.) in DMF to the beads using HCTU (4 eq.), and NMM (8 eq.) for 1 h. The Fmoc protection group was cleaved off by 20% piperidine in DMF for 30 min. Beads were washed thoroughly with DMF after each step.

### 2.5. Peptide Ladder Synthesis 

For the synthesis of the peptides, Fmoc-amino acids (0.1 mol/L, 4 eq.) and Boc-amino acids (0.2 eq.) in DMF were coupled to the prepared resin using HCTU (4 eq.), and NMM (8 eq.) for 1 h. Fmoc was cleaved off by 20% piperidine in DMF for 30 min. The monitoring of the dibenzofulvene-piperidine adducts during the peptide synthesis revealed that between 3% and 10% Boc-protected amino acids were incorporated during every coupling step. On average, 60%–80% of the peptides on the surface are full-length peptides (Supplement, Figure S7). Beads were washed thoroughly with DMF after each step. The side chains and Boc-truncated peptides were deprotected by treatment with trifluoroacetic acid (TFA)/triisopropylsilane (TIS)/H_2_O (95/2.5/2.5 v/v/v) twice for 2 h, respectively. 

### 2.6. Synthesis of the Split-and-Mix One-Bead-One-Compound Peptide Library

The fully randomized peptide library was synthesized on the prepared resin by following ladder synthesis protocol by manually splitting the resin in aliquots. Subsequently, all resin aliquots were mixed for washing, acetylation, and Fmoc deprotection. The cycle of splitting and mixing was repeated until the desired peptide length was accomplished. Finally, the resin was treated with a mixture of 95% trifluoroacetic acid (TFA), 2.5% triisopropylsilane (TIS), and 2.5% deionized water to remove side chain protecting groups, followed by intensive washing with DCM and drying of the resin in vacuo. 

### 2.7. Surface Plasmon Resonance (SPR) Experiments 

Surface plasmon resonance (SPR) experiments were performed on a Reichert SR7500DC dual channel SPR system. A total of 100 µL of 100 μg/mL of the antibody in 1 mM sodium acetate (pH 5.0) was immobilized onto one flow channel of the biosensor chip HC200 (XanTec bioanalytics, Düsseldorf, Germany) using a standard amine coupling protocol (Supplement, SPR chip preparation) [29]. Peptide stock solutions (1 and 10 mM) were prepared in HBS-EP (pH 7.4, 0.01 M 4-(2-hydroxyethyl)-1-piperazine ethanesulfonic acid (HEPES), 0.15 M NaCl, 3 mM EDTA, 0.005% Tween 20) and later diluted in HBS-EP buffer. Peptide binding was performed in a multiple kinetics mode using HBS-EP as a running buffer. Binding kinetics parameters were calculated using TraceDrawer Data Analysis Software.

More details of most experiments can be found in the Supplementary Materials.

## 3. Results

### 3.1. High-Throughput Screening

A peptide library with a length of eight amino acids was synthesized on a TentaGel HL NH_2_ (75 µm) resin, which is suitable for both peptide synthesis in organic media and biochemical assays in aqueous buffers. The peptides were attached to the resin via a base-labile HMBA-linker, which is compatible with Fmoc chemistry and does not interfere with the MS analysis after cleavage. 

To achieve a high-throughput method, a screening process based on a fluorescence read-out with a microarray scanner and peptide identification via MALDI-TOF MS was performed on the same chip. These glass-based chips were prepared by modifying the surface of a microscope slide with an electrically conductive, double-sided adhesive tape. A metal sieve with a mesh of 100 µm was pushed on the surface of the adhesive tape. The dried beads were poured on the sieve and manually spread to allow the beads to fill the meshes. Slight pressing on top of the resin beads ensured their firm attachment on the sticky surface. The removal of the grid resulted in an ordered layer of beads on the chip surface, where every bead has a defined distance to the adjacent beads (Supplement, Figure S1). Between 30,000 and 50,000 beads can be immobilized on one chip using this procedure.

**Figure 1:**
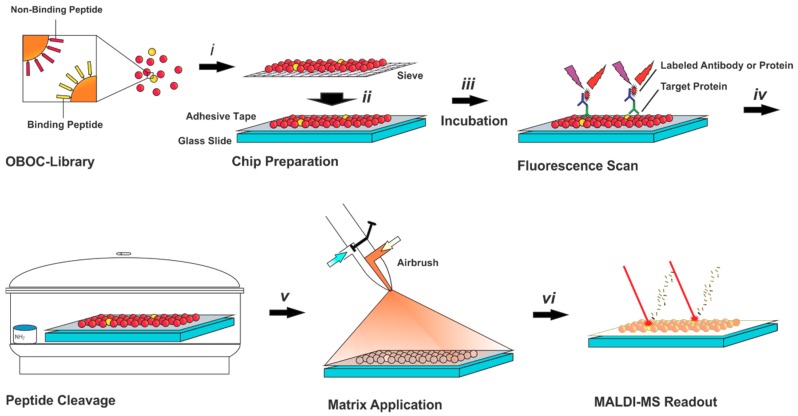
Illustration of the on-chip screening for peptide-based protein binders. The chip is prepared by sieving the one-bead-one-compound (OBOC)-library (i) on a glass slide covered with an electrically conductive, double-sided adhesive tape (ii). On-chip incubation with the target protein and a labeled secondary antibody is performed (iii), followed by a high-resolution fluorescence scan for the identification of protein binding peptides. For peptide cleavage, the chip is placed in a chamber with ammonia vapor (iv). using matrix-assisted laser desorption/ionization (MALDI) matrix is applied with an airbrush (v) and the sequence read-out of positive peptides is achieved via ladder sequencing by MALDI-time of flight (TOF) mass spectrometry (MS).

All reaction steps were performed directly on the chip by incubating with the labeled target molecules. The beads, displaying target-binding peptides, were identified by a high-resolution fluorescence scan using a suitable wavelength corresponding to the fluorescence label of the target (Figure 1). 

For sequence determination on the same chip, the peptides were released by placing the slide in a chamber with gaseous ammonia for a minimum of two hours. Matrix application was performed using a conventional airbrush gun. Under a light microscope, a thin layer of the matrix is visible on the surface of the beads (Supplement, Figure S2). No cross contamination of the peptides was observed.

### 3.2. Ladder Sequencing

**Figure 2:**
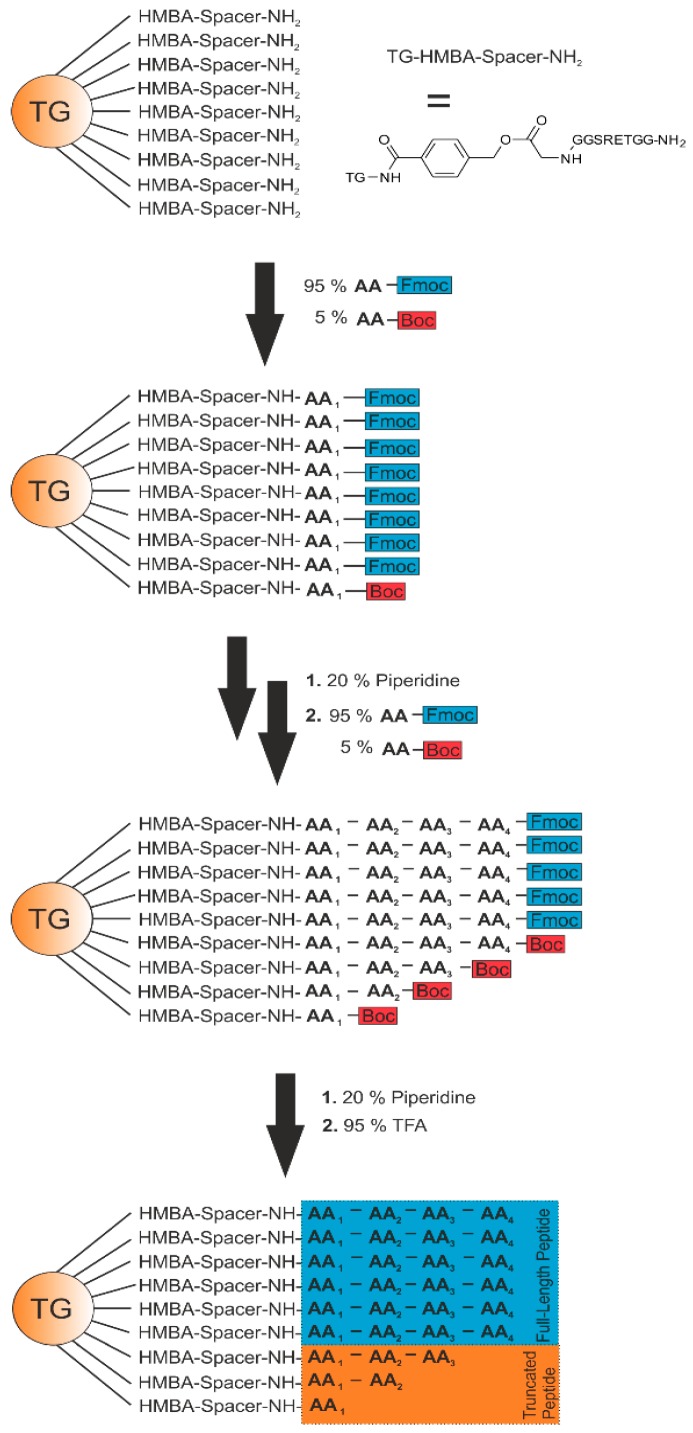
Scheme of the generation of a ladder sequence. As solid support, a TentaGel (TG) resin (approximately 75 µm) is used. The base-labile linker 4-hydroxymethylbenzoic acid (HMBA) and a spacer sequence are attached to the resin using a standard Fmoc protocol. The synthesis is performed by using 95% of Fmoc-AA and 5% of Boc-AA. Deprotection of the Fmoc-AA is performed by using 20% piperidine in dimethylformamide (DMF) after every coupling step. Permanent side chain and Boc protection groups are removed by 95% trifluoroacetic acid (TFA).

De novo sequencing in general, as already described, is a challenging task. A ladder sequencing approach was established based on the technique of St. Hilaire et al. [23]. In contrast to the previously described methods, not 10%, but only 5% of the Boc-protected amino acids were added as truncation agents in each coupling step (Figure 2), ensuring that the majority of peptides on the surface contain the full-length sequence.

The beads containing positive peptides were identified by fluorescence imaging and their coordinates were transformed into coordinates for the MALDI mass spectrometer. After peptide cleavage by ammonia vapor and subsequent matrix application, the peptides were sequenced by MALDI-TOF MS directly on the bead surface without any manual manipulation. The ladder-like peaks allowed an easy structural sequence assignment. As this sequencing method mainly relies on the mass difference between the peaks, corresponding to the molecular weight of the respective amino acid building block, this method is less dependent on precise external calibration, which may be time-consuming and inconvenient.

Though the method leads to truncated peptide species on the beads, it can be expected that the shorter peptides do not interfere with the screening process in most cases. On the one hand, they are present in a relatively low concentration and on the other hand, shorter peptides can be assumed and are reported to show lower affinities than their full-length counterparts [30].

To establish an unbiased and high-throughput method for the evaluation of peptide truncation spectra, an algorithm was developed allowing for their automated processing [31]. This provides a convenient tool for the decoding of the sequences from the MALDI-TOF MS. In the software tool, the most abundant peak is defined as the full-length peptide. The peaks at lower m/z are assigned automatically and compared to the theoretical ladder sequences of all peptides from the underlying peptide library. The peptide sequences are sorted by the number of matching amino acids. For parallel analysis, a batch process is included, allowing for the high-throughput processing of multiple spectra.

### 3.3. Prescreening

**Figure 3:**
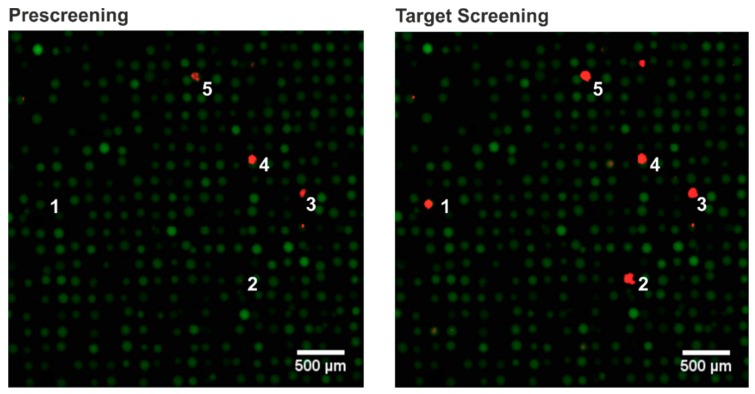
Fluorescence scans (resolution: 10 × 10 µm^2^ per pixel) of an OBOC library performed as on-chip screening. Overlay of green (excitation 532 nm, emission 570 nm) and red (excitation 635 nm, emission 670 nm) fluorescence. Green fluorescence was used to detect all TentaGel beads utilizing their auto-fluorescence and red fluorescence was used to identify positive beads. Left: prescreening with the secondary labeled antibody (red fluorescence dye) for false positive identification. Right: target screening with the target protein followed by secondary antibody. The beads with the numbers 1 and 2 show a significantly higher fluorescence intensity and were hence considered as “hits”. In contrast, the beads with the numbers 3, 4, and 5 show similar intensities in the prescreening and target screening and were flagged as false positives.

The screening of an OBOC library often requires a secondary protein that is labeled with a fluorophore or enzyme. For example, secondary antibodies [5] or streptavidin for staining biotinylated proteins have been used [32]. In these cases, false positive peptides might be identified that show affinity to the secondary reagent and not to the target protein. Furthermore, it is known that occasionally some defective TentaGel beads show a high autofluorescence [33]. These false positive hits are a major technical issue in OBOC libraries. Ding et al. suggested identifying these beads prior to the screening to remove these beads manually [34]. In our chip-based technique, manual elimination is not necessary. The bead-coated chip can be examined by a high-resolution fluorescence scan in advance. Thus, any beads showing a high autofluorescence can be identified and flagged. Furthermore, the chip can be incubated solely with the secondary fluorophore-labeled antibody without the target to identify any false positives binding to the secondary reagent. Regeneration of the chip by denaturing reagents such as urea or guanidine hydrochloride can be used to remove bound reagents. The fluorescence intensities from the target screening and the prescreenings were compared. Only if the detected intensity of a single bead in the target screening was three times higher than in the prescreening, the bead was considered as a “hit” (Figure 3).

### 3.4. Screening Validation

**Figure 4:**
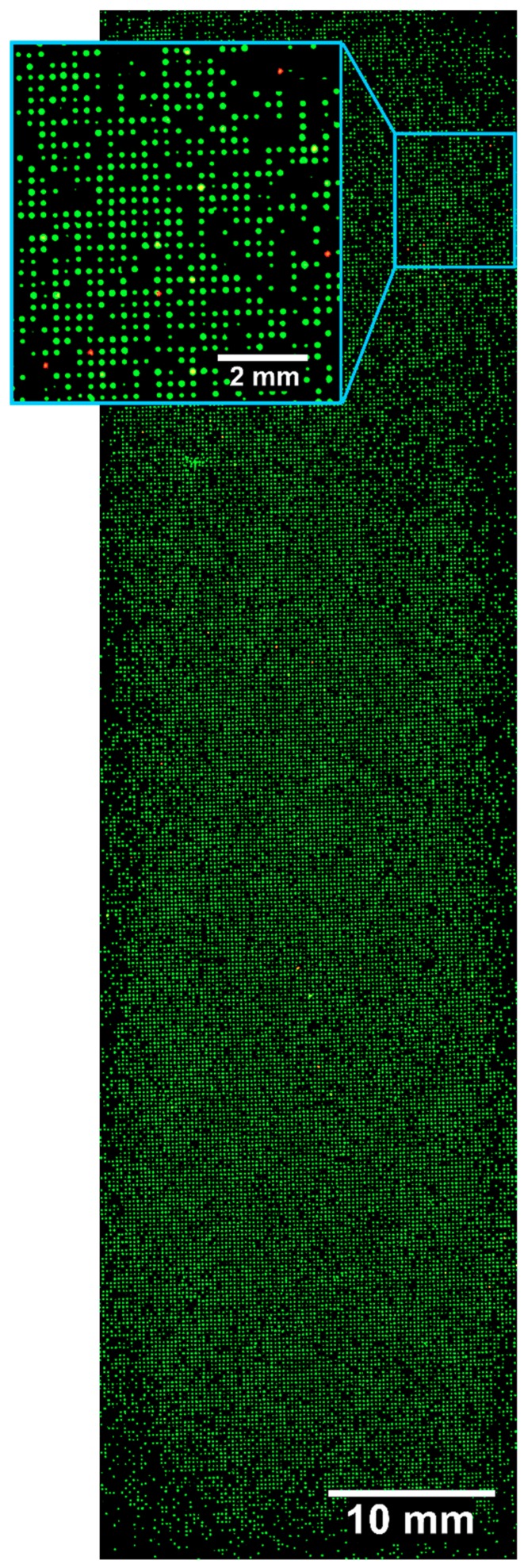
High-resolution fluorescence scan (10 × 10 µm^2^ per pixel) of a chip with about 33,000 immobilized beads after incubation with anti-FLAG antibody and staining with anti-mouse IgG Atto633. Overlay of green (excitation 532 nm, emission 570 nm) and red (excitation 635 nm, emission 670 nm) fluorescence. Green fluorescence is used to locate all TentaGel beads utilizing their auto-fluorescence and red fluorescence is used to identify FLAG-displaying beads.

To examine and validate the technical aspects of the presented screening technique, the well-established FLAG peptide [35] in combination with the monoclonal mouse anti-FLAG antibody M2 was chosen [36]. Four peptides were separately synthesized on TentaGel beads, from which one peptide contained the known FLAG sequence and three others contained the random peptides. The four model peptides were synthesized using the described ladder synthesis, theoretically resulting in 66% of the full-length peptide and 34% of truncation sequences. Fifty FLAG peptide-displaying beads were doped into about 50,000 beads displaying random peptides, which had been tested to show no significant interaction with the M2 anti-FLAG antibody. Experiments were performed to test the efficiency with which the known number of FLAG peptide-displaying beads could be identified from a mock library. A total of 33,000 of the 50,000 beads could be immobilized on a single slide. The slide with the immobilized beads was incubated with the anti-FLAG antibody, followed by washing and staining with the fluorescent anti-mouse antibody (Atto633 conjugate). A total of 35 out of about 33,000 beads could be identified as binders by a high-resolution fluorescence scan (Figure 4). The sequence identification was performed directly on the same chip as described above. The peptides were cleaved from the beads, the matrix was applied on the chip, and the coordinates of the FLAG antibody binding beads were transformed for MALDI-TOF MS and directly measured on the bead surface. The ladder-like sequence peaks were obtained, allowing a structural assignment, and all 35 peptides were identified as FLAG peptides.

### 3.5. Validation of the Ladder Sequencing

The robustness of the ladder sequencing was first tested by the automatic sequence determination of the four model peptides. Therefore, three FLAG peptides and three of each of the non-FLAG peptides were directly examined on the bead surface. They were easily identified by the parent mass and the calculated sequence compared with the expected sequence. Overall, 100% of the amino acids were identified at the correct position in the sequence (Supplement, Tables S5–S8).

**Figure 5:**
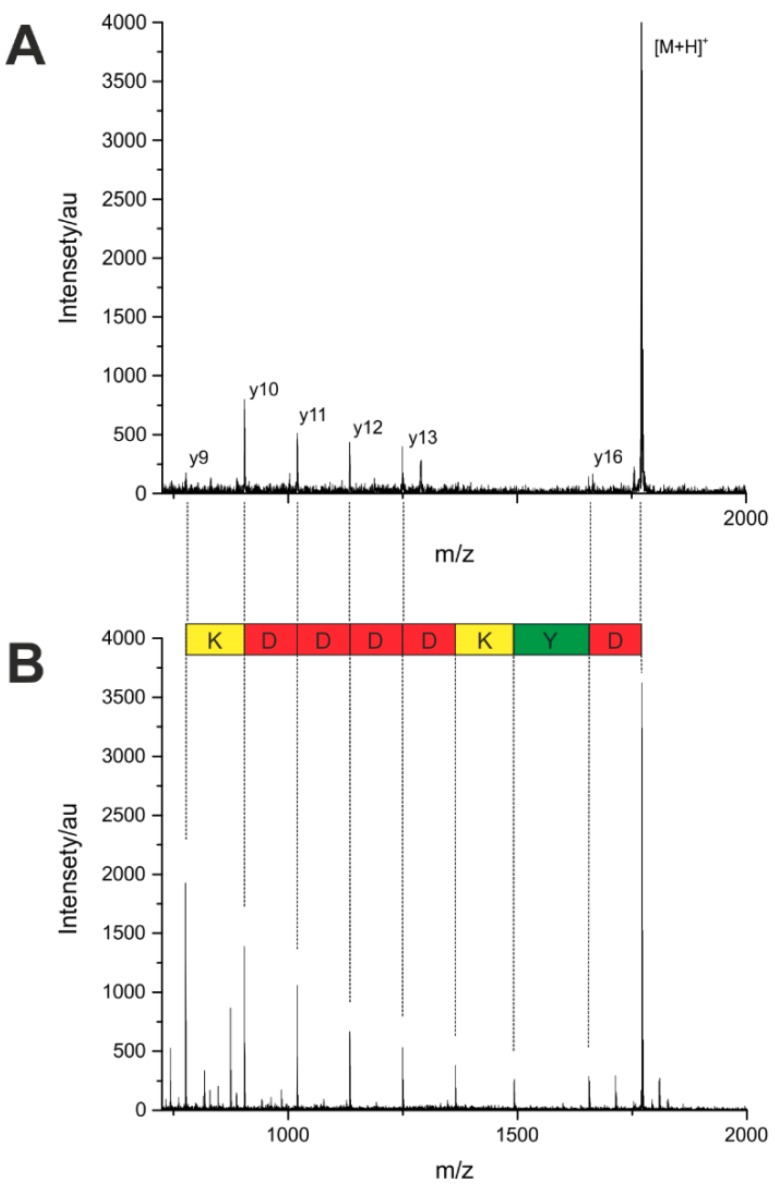
(**A**) Fragmentation-based MALDI-TOF MS/MS of a peptide from a single bead and interpretation of the tandem mass spectrum. (**B**) Ladder sequencing of the same peptide by MALDI-TOF MS.

To compare the sequencing by MS/MS fragmentation from a single bead with the ladder sequencing, the most abundant peaks from the measured peptides were fragmented. The parent ion mass was detected in high intensities, the detection of some fragment ions, on the contrary, was difficult, if not impossible. As shown in Figure 5, the intensity of the fragment ions is low in comparison with that of the parent ion, and several cleavage sites are missing completely. This lack of information leads to an incomplete sequence assignment. One reason for the poor fragmentation efficiency, in addition to the aforementioned problems, is the very small sample amount of a single bead. A custom-made search tool was used, automatically identifying the corresponding peptide sequences. The results are summarized in Table 1, showing that only 29% of the amino acids within an individual sequence were recognized correctly by MS/MS (Supplement, Tables S1–S4). 

### 3.6. Targeted Screening for Anti-FLAG Antibody Ligands

To explore the potential of the developed method, a targeted peptide library was synthesized for the identification of peptides binding to the FLAG antibody M2. This clone binds to a well-studied epitope, consisting of the amino acids DYKXXD [37].

For the library synthesis, the five L-amino acids threonine, isoleucine, aspartic acid, lysine, and tyrosine were used. The library was synthesized using conventional split-and-mix synthesis. The cycle of splitting and mixing was repeated eight times, resulting in a theoretical diversity of 390,625 peptides. A chip was prepared with about 12,000 immobilized beads in total. Prescreening was performed, in which approximately 10% of the positive beads were identified as false positives. It can be assumed that in a larger and more complex library, the fraction of false positive beads may be even higher (Scans in Supplement, Figure S3). 

The chip was then incubated with the primary anti-FLAG antibody for 15 h, followed by incubation with the secondary antibody for 1 h. The chip was analyzed by high-resolution fluorescence scan. Thirty-three beads with the highest fluorescence were identified as anti-FLAG antibody binding peptides and selected for ladder sequencing. All sequences are listed in the Supplement. Most selected sequences showed either the epitope DYKXXD (10) or partial epitopes with three out of the four amino acids of the epitope (14). Eight of the sequences contain less or no obvious parts of the epitope (all sequences in Supplement, Table S9). 

For characterization, three of the positive peptides were resynthesized—Peptide 1 (DYKDYDKD) containing the known epitope and Peptide 2 (DYIDYDYK) representing a partial epitope. Peptide 3 (DYDIYIYD) was created as a consensus peptide by choosing the most abundant amino acid at every position from 32 identified positive peptides.

Surface plasmon resonance (SPR) and on-bead fluorescence assays were performed to estimate the binding affinities of the given peptides. In the fluorescence assays, the beads with the respective peptides were incubated with the anti-FLAG antibody and stained with the fluorescent secondary antibody. For the SPR experiments, the anti-FLAG antibody M2 was immobilized on an HC200 chip and peptide solutions from 6 nM to 1 µM were examined. The results are presented in Table 2 (Supplement, Figure S5). 

### 3.7. Non-Targeted Screening for IgG Ligands

Peptides binding specifically to IgG are of special interest in the emerging market of monoclonal antibodies for diagnostic and particularly therapeutic purposes [39]. The isolation of antibodies from complex sera or cell culture media is usually performed by affinity columns with immobilized protein A. The advantage of using peptides in contrast to proteins is that they can be produced synthetically and do not contain any impurities from a microbiological origin. Additionally, they are much smaller than proteins, allowing a higher ligand density on the affinity column.

For the library synthesis, the eight amino acids, serine, isoleucine, glycine, glutamic acid, arginine, phenylalanine, proline, and histidine, were used. The total length of seven amino acids per peptide results in a theoretical library size of 2,097,152 different peptides. A chip was prepared by immobilization of about 33,000 beads. The screening was performed using polyclonal mouse IgG and an anti-mouse IgG Atto633 for staining (Scans in Supplement, Figure S4). Nine peptides were identified as antibody binding and their sequence was successfully determined by ladder sequencing (Supplement, Table S10). Three of the identified peptides were synthesized and their affinities were analyzed by SPR. The measured affinities are in the micromolar range (Table 3; Supplement, Figure S6). 

## 4. Discussion

The presented on-chip screening technique was developed by modifying a glass-based microscopy slide with double-sided conductive tape. The beads are immobilized spreading them through a sieve, which is an easy and cost-effective alternative to complex fabrication processes used previously for other chips [9]. The screening was successfully validated by utilizing a FLAG-based model system. It was shown that screening and sequence identification is possible on the same chip. This allows for the rapid and reliable identification of peptides as protein binders without any manual bead picking or manipulation.

Thirty-five out of 50 positive peptide beads could be recovered from a mock library of 50,000 beads in total. During the whole process, no cross contamination by peptides from neighboring beads was observed. 

The loss of 15 positive beads can be rationalized by the limitations of the immobilization procedure. Because of the soft polyethylene glycol surface of TentaGel beads, about one-third of the beads stuck on the utilized sieve and were not immobilized on the modified chip. In the screening of real libraries, the losses are not very relevant, because primarily, the overall number of beads screened is important in OBOC libraries.

The occurrence of false positive hits is by far the severest limitation of combinatorial libraries. The novel and robust chip format allows for repeated regeneration of the chip surface by denaturation of bound proteins. This opens the possibility for multi-step incubations and control scans, highly reducing the number of false positive hits. This is an additional improvement to the chip-based technologies presented previously [9]. 

For the challenging de novo sequencing by MALDI-TOF MS, a powerful encoding strategy using a peptide ladder synthesis was established. The results presented in Table 1 clearly show that the ladder sequencing technique results in significantly better sequence reliability in comparison with the classical fragmentation technique.

The technique was tested by screening a targeted library against anti-FLAG antibody M2 and 33 peptides were identified as positive. One-third of the peptides contain the known epitope for the anti-FLAG antibody. The rest of the identified sequences display parts of the epitope or no previously reported epitopes. These are designated as mimotopes—peptides which mimic the structure of an epitope [41]. Three of these newly identified peptides were characterized with on-bead incubation by fluorescence assay and SPR (Table 2). The relative fluorescence intensities measured on the beads are in acceptable agreement with the values determined with SPR. Peptide 1, which contains five amino acids identical to the known FLAG sequence, shows affinities comparable to the literature value of 31 nM [38]. The mimotope Peptide 2 shows significantly lower affinities. Interestingly, the consensus Peptide 3 shows one of the highest affinities of the respective peptides, without containing any reported epitope for the anti-FLAG antibody M2. This demonstrates that this screening technique unbiasedly identifies protein binders, revealing new peptide binders for the anti-FLAG antibody M2.

In a non-targeted approach, a library was screened against mouse immunoglobulins, resulting in several peptides with a significant binding property. Four of these peptides were resynthesized and characterized by SPR, showing micromolar affinities (Table 3). For linear peptides of this size, affinities in this range are in good agreement with the literature [42]. 

## 5. Conclusions

An on-chip technique was developed, which is comprised of an OBOC peptide library immobilized on a surface, followed by protein-binding assays based on high-resolution fluorescence detection and direct on-bead peptide sequencing by mass spectrometry. The “all-on-one chip” system eliminates tedious separation or manual picking steps and may greatly facilitate the discovery of protein binders from peptide libraries. Furthermore, the novel chip format allows multi-step incubations, which opens the possibility for control scans, highly reducing the number of false positive hits. Finally, the technique was tested successfully by screening a targeted and a non-targeted peptide-library, revealing new peptide binders for the anti-FLAG antibody M2 and mouse immunoglobulins. 

The presented chip-based technique can be adapted further for multiple application of OBOC libraries. Using a comparable strategy peptide-PEG conjugates for the solubilization of the drug BB17 could be identified [26].

By combining this approach with a prior magnetic enrichment strategy as described by Mendes et al. [5], the library size might be increased even further. Additionally, if the diameter of the beads could be reduced, a significantly higher number of immobilized beads per chip could be achieved.

## Figures and Tables

**Table 1:** Performance of the conventional fragmentation approach (tandem mass spectrometry (MS/MS)) in comparison with the ladder sequencing approach (MS). The number of residues correctly identified is shown, as is the total number of residues within the sequence. Fragmentation and ladder sequencing were performed from a single bead.

Parent Mass (m/z)	Peptide Sequence with Linker GGTERSGG	AA Recognized by MS/MS Fragmentation		AA Recognized by Ladder Sequencing	
		% Correct Residues	% Correct Position	% Correct Residues	% Correct Position
**1700.75**	DYKDDDDK	46	17	100	100
**1600.76**	DTHFPIGG	63	20	100	100
**1586.73**	VPFHTDGG	50	33	100	100
**1737.94**	KFVPFKKS	62	16	100	100

**Table 2:** Sequences of the FLAG peptide and three resynthesized positive peptides. The relative fluorescence determined by on-bead incubation with the anti-FLAG antibody, setting the fluorescence intensity of the FLAG peptide to 100%. Equilibrium dissociation constant (K_D_) measured by surface plasmon resonance (SPR).

Peptide	Sequence	Relative Fluorescence	K_D,_ nM
**FLAG**	DYKDDDDK	100%	50 ± 30/31 [38]
**Peptide 1**	DYKDYDKD	97%	15 ± 5
**Peptide 2**	DYIDYDYK	37%	240 ± 60
**Peptide 3**	DYDIYIYD	73%	12 ± 5

**Table 3:** Equilibrium dissociation constants (K_D_) for peptide mouse IgG interaction determined by SPR.

Peptide	Sequence	K_D,_ µM
**IgG BP 1**	GRIHGPR	14 ± 1
**IgG BP 2**	GIHPFGR	50 ± 10
**IgG BP 3**	GEPFSIP	20 ± 30
**IgG BP 4**	SHSGIFR	60 ± 5
**[40]**	HWRGWV	10
**[39]**	HFRRHL	26

## Supplementary Materials

The following are available online at https://www.mdpi.com/2571-5135/8/2/11/s1. 1. Materials, 2. Resin preparation, 3. Peptide synthesis, 4. Synthesis of peptide library 1, 5. Synthesis of peptide library 2, 6. Chip preparation, 7. Cleavage of peptides and matrix-application via airbrush gun, 8. Sequencing of test peptides by MALDI-TOF MS/MS (fragmentation), 9. Ladder-sequencing of test peptides by MALDI-TOF MS, 10. Incubation with anti-FLAG antibody M2, 11. Ladder sequencing of the peptides selected from library 1 after anti-FLAG antibody incubation, 12. Ladder sequencing of the peptides selected from library 2 after polyclonal mouse IgG incubation, 13. Peptide ladder-sequencing by MALDI TOF MS/MS of library 2, 14. SPR chip preparation, 15. SPR characterization of anti-FLAG binding peptides, 16. SPR characterization of mouse IgG binding peptides.
