# Supplementary material for: Efficient Screening of Combinatorial Peptide Libraries by Spatially Ordered Beads Immobilized on Conventional Glass Slides
Source: High Throughput. 2019 Apr 30;8(2):11. doi: 10.3390/ht8020011 (PMC6631230; doi:10.3390/ht8020011)
Supplement: Supplementary file 1 [file high-throughput-08-00011-s001.pdf]

Supplementary Material: Efficient Screening of Combinatorial Peptide Libraries by Spatially Ordered Beads Immobilized on Conventional Glass Slides

Timm Schwaar [1,2], Maike Lettow [1,3], Dario Remmler [1,2], Hans G. Börner [2] and Michael G. Weller [1,*]

[1] Division 1.5 Protein Analysis, Federal Institute for Materials Research and Testing (BAM), Richard-Willstätter-Strasse 11, 12489 Berlin, Germany; timm.schwaar@bam.de (T.C.); maike.lettow@fu-berlin.de (M.L.); dario.remmler@chemie.hu-berlin.de (D.R.)

[2] Department of Chemistry, Humboldt-Universität zu Berlin, Brook-Taylor-Straße 2, 12489 Berlin, Germany; boernerh@hu-berlin.de

[3] Institut für Chemie und Biochemie der Freien Universität Berlin, Takustraße 3, 14195 Berlin, Germany

List of Contents

List of Contents 1

1. Materials 1

2. Resin Preparation 2

3. Peptide Synthesis 2

4. Synthesis of Peptide Library 1 3

5. Synthesis of Peptide Library 2 3

6. Chip Preparation 3

7. Cleavage of Peptides and Matrix-application *via* Airbrush Gun 4

8. Sequencing of Test Peptides by MALDI-TOF MS/MS (fragmentation) 5

9. Ladder-sequencing of test peptides by MALDI-TOF MS 6

10. Incubation with Anti-FLAG Antibody M2 8

11. Ladder Sequencing of the Peptides Selected from Library 1 after Anti-FLAG Antibody Incubation 9

12. Ladder Sequencing of the Peptides Selected from Library 2 after Polyclonal Mouse IgG Incubation 10

13. Peptide Ladder-sequencing by MALDI TOF MS/MS of Library 2 11

14. SPR Chip Preparation 12

15. SPR Characterization of Anti-flag Binding Peptides 12

16. SPR Characterization of Mouse IgG Binding Peptides 13

17. Monitoring of Dibenzofulvene-Piperidine Adducts in Ladder Synthesis 14

References 14

1. Materials

The solvent dimethylformamide (DMF, 1.00387) was purchased from *Merck* (Darmstadt, Germany), ethanol 99.8% for analysis, absolute (493511), from *Sigma Aldrich* (St. Louis, MI, USA) and

dichloromethane (2356) from *Th. Geyer GmbH & Co. KG* (Renningen, Germany). Lab water was obtained from a Milli-Q water purification system (*Millipore*, Bedford, MA, USA) with a resistivity of > 18.2 Ω and TOC-value of < 5 ppb. The resin for peptide synthesis TentaGel HL NH_2 (HL12902) was purchased from *Rapp Polymere GmbH* (Tübingen, Germany). The linker 4-hydroxymethylbenzoic acid (HMBA, AB131645) was obtained from *abcr* (Karlsruhe, Germany). The coupling reagents diisopropyl carbodiimide (DIC, D125407-5G) and 4-methylmorpholine (NMM, M56557) were purchased from *Sigma Aldrich.*

The protected amino acids Fmoc-Thr(tBu)-OH, Fmoc-Gly-OH, Fmoc-Ser(tBu)-OH, Fmoc-Pro-OH and Fmoc-Phe-OH were purchased from *Bachem* (Bubendorf, Switzerland); Fmoc-Asp(OtBu)-OH and Fmoc-Ile-OH from *Carl Roth GmbH & Co. KG* (Karlsruhe, Germany); Fmoc-Arg(Pbf)-OH, Boc-Arg-OH, Boc-Glu(OtBu)-OH and Boc-Ser-OH from *J&K Chemical Ltd.* (Shanghai, China); Fmoc-Tyr(tBu)-OH, Fmoc-His(Trt)-OH, Fmoc-Glu(OtBu)-OH, Boc-Phe-OH, Boc-Pro-OH, Boc-Tyr-OH and Boc-Lys(Boc)-OH from *Sigma Aldrich*; Fmoc-Lys(Boc)-OH, Boc-Gly-OH, Boc-His(Tos)-OH and Boc-Ile-OH from *IRIS Biotech GmbH* (Marktredwitz, Germany). The coupling reagent O-(1H-6-Chlorobenzotriazole-1-yl)-1,1,3,3-tetramethyluronium hexafluorophosphate (HCTU, 4256.2) was purchased from *Carl Roth GmbH & Co. KG* (Karlsruhe, Germany). For deprotection, triisopropylsilane (TIS, 233781) and trifluoroacetic acid (TFA, T6508) were purchased from *Sigma Aldrich.*

The monoclonal anti-FLAG M2 antibody produced in mouse, 1 mg/mL, clone M2, affinity isolated antibody, buffered aqueous solution (F1804-50UG), anti-Mouse IgG-Atto 633 antibody produced in goat (78102-1ML-F), Bovine Serum Albumin (A7906) and polyclonal mouse IgG antibody (Protein A purified, PP54) were purchased from *Sigma Aldrich*.

The chemicals Tween 20 (P7949) HEPES (H4034-100G), NaCl (71376), EDC (E1769), ethanolamine (02400), guanidine hydrochloride (G3272-25G), ammonia water (28–30%, 221228) were obtained from *Sigma Aldrich*. PBS buffer (1X, Dulbecco's) (A0964) EDTA (131669) were purchased from *AppliChem GmbH* (Darmstadt, Germany). The MALDI matrix 2,5-dihydroxyacetophenone (DHAP, A12185) from *Alfa Aesar* (Ward Hill, USA) from Sigma. MES (4256.2) was obtained from *Carl Roth GmbH & Co. KG (Germany).*

2. Resin Preparation

TentaGel HL NH_2 resin was swollen in DMF for 1 h and washed with DMF. HMBA (0.1 mol/L, 4 eq), HCTU (7.2 eq) and NMM (16 eq) were dissolved in DMF and added to the resin for 1 h. The HMBA coupling was repeated using identical amounts of each reagent. The resin was washed and subjected to the Kaiser test to confirm that the reaction had gone to completion [1].

For the incorporation of the first amino acid, Gly-Fmoc (3 mol/L, 10 eq) and DIC (5 eq) were dissolved in DCM and stirred at 2°C for 20 min. The mixture was added to the resin, followed by dropwise addition of DMAP (0.1 eq, 50 mmol/l in DMF). After 10 h, the resin was washed with DMF and DCM. Coupling was repeated once overnight. Fmoc was cleaved off by 20% piperidine in DMF for 30 min. The coupling efficiency was determined by screening of the deprotection solution. This was performed using an Evolution 220 UV-Visible Spectrophotometer by *Thermo Fisher Scientific* (*Massachusetts, USA*), recording the specific absorption of the dibenzofulvene-piperidine adduct at 301 nm. The yield was about 60 %.

3. Peptide Synthesis

The spacer sequence GGTERSG for the peptide libraries, test peptides (DYKDDDDK, DTHFPIGG, VPFHTDGG, KFVPFKKS) and resynthesized peptides from screening (GRIHGPR, GIHPFGR, GEPFSIP, SHSGIFR) were manually synthesized by adding Fmoc-amino acids (0.1 mol/L, 4 eq.) in DMF to the resin using HCTU (4 eq.), and NMM (8 eq.) for 1 h. Fmoc was cleaved off by 20% piperidine in DMF for 30 min. Beads were washed thoroughly with DMF after each step. Fmoc monitoring of the deprotection solution was performed by UV/VIS spectrometer. Finally, the resin was treated with a mixture of 95 % TFA, 2.5 % TIS, 2.5 % lab water to remove side chain protecting groups, followed by intensive washing with DCM and drying of the resin *in vacuo*.

4. Synthesis of Peptide Library 1

The library was synthesized using 20 mg of a TentaGel HL NH_2 resin modified with HMBA and the Spacer sequence GGTERSG. The variable peptide segment/peptide library was synthesized manually by splitting the resin into eight equal aliquots. Coupling of each aliquot was performed using 8 eq. of each Fmoc protected amino acid (Thr, Ile, Asp, Lys, Tyr) and 0.4 eq of the corresponding Boc-protected amino acid in DMF with HCTU (4 eq.), and NMM (8 eq.) for 1 h or overnight. All aliquots were mixed for washing and Fmoc deprotection. This cycle of splitting and mixing was repeated eight times. Finally, the resin was treated with a mixture of 95 % TFA, 2.5 % TIS, 2.5 % lab water to remove side chain protecting groups and Boc protecting groups from the truncated peptide sequences, followed by intensive washing with DCM and drying of the resin *in vacuo*.

5. Synthesis of Peptide Library 2

The library was synthesized using 500 mg of a TentaGel HL NH_2 resin modified with HMBA and the spacer sequence GGTERSG. The variable peptide segment/peptide library was performed as described before using 8 eq. of the respective Fmoc-protected amino acids (Gly, His, Ile, Ser, Phe, Pro, Glu, Arg) and 0.4 eq (5 %) of the corresponding Boc-protected amino acids.

6. Chip Preparation

A plain microscope slide (J. Melvin Freed) with a size of 75 × 25 mm^2 was modified by attachment of the 3M XYZ-Axis electrically conductive double-sided adhesive Tape 9713, which is an isotropically conductive, pressure sensitive tape. The tape conducts electrically through the thickness (Z-axis) and in the plane of the adhesive (X, Y planes) and is ideal for MALDI-TOF MS/MS measurements. It contains a nickel-plated carbon scrim. The modified microscope slide was fixed temporarily in a custom-made slide holder. A sieve made from stainless steel with a mesh of 100 µm was attached on the surface of the adhesive tape. The dried beads were spread on the sieve and gently dispersed to allow the resin to fill the meshes. Slight pressing on top of the beads ensured attachment on the sticky surface.

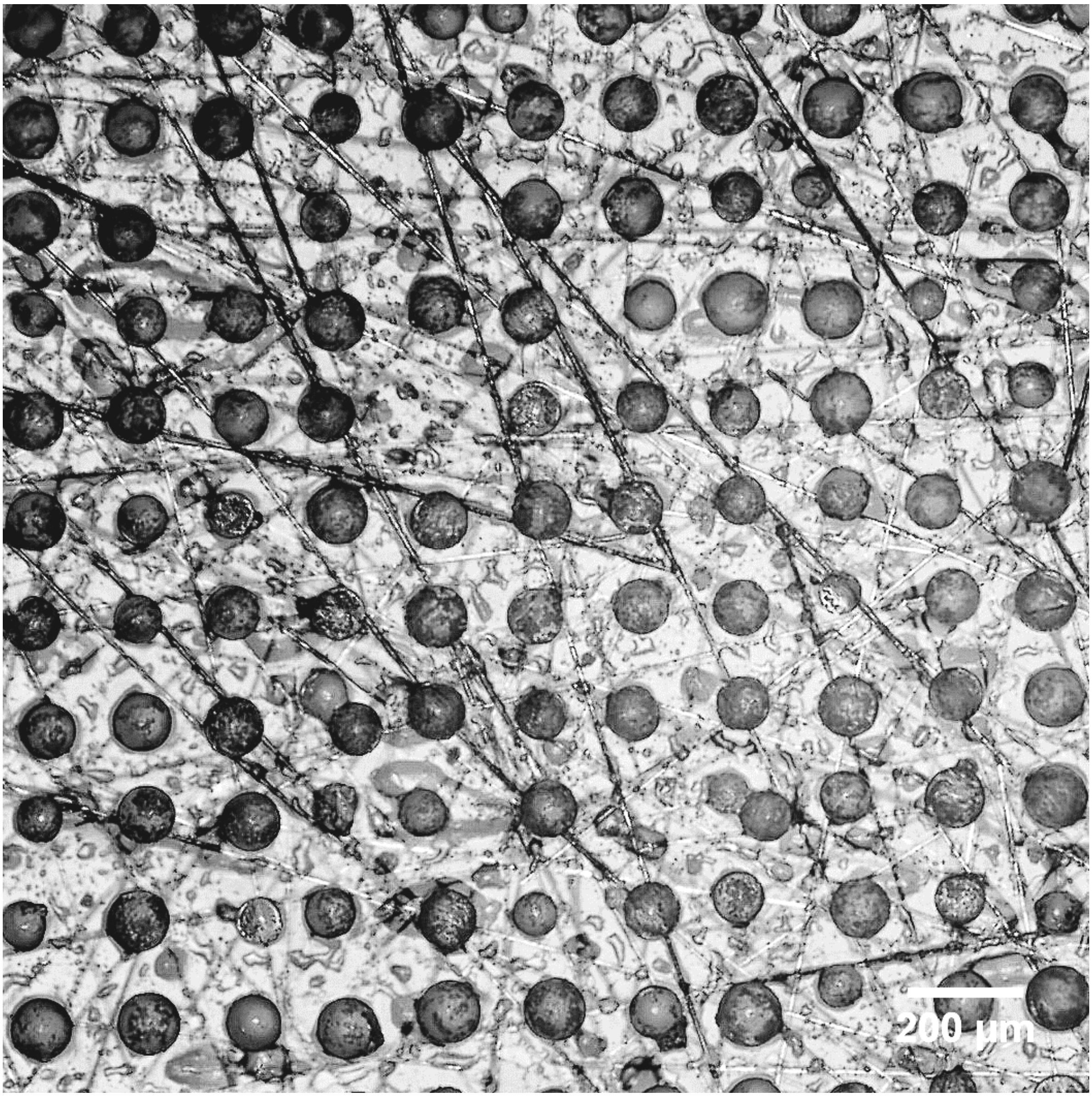

Figure S1. of a layer of 75 µm TentaGel beads, immobilized on a surface of a microscope slide modified with an electrically conductive, double-sided adhesive tape. Small nickel wires are visible in the background, responsible for the electrical conductivity.

7. Cleavage of Peptides and Matrix-application *via* Airbrush Gun

The incubation was followed by regeneration of the chip. To remove all salts, the chip was extensively washed with lab water and dried. For peptide cleavage from the bead surface, the chip was placed in a chamber with 2 ml of aqueous ammonia solution (30%) at the bottom. The linker was efficiently cleaved within 2 h. Matrix application was achieved using a conventional Agora-Tec airbrush (Airbrush Pistole Kit AT-AK-01, *Agora-Tec*, Schmalkalden, Germany). 2 mL of matrix solution was used per chip, consisting of 20 mg DHAP in ethanol, dibasic ammonium citrate (18 mg/mL in lab water) (3:1 v/v). The chip was placed 15 cm under the tip of the airbrush and a constant pressure of 2 bar was applied to the airbrush gun. This ensured the formation of a thin matrix layer on the surface of the bead (Figure S2).

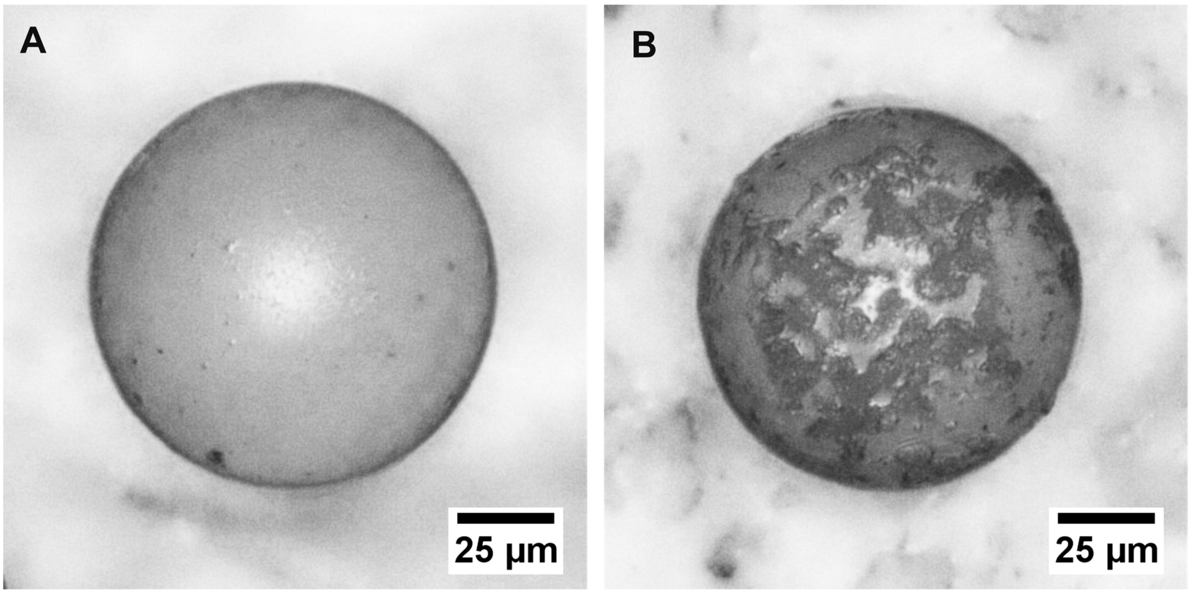

Figure S2. Immobilized TentaGel beads (**A**): Before matrix application, (**B**): After matrix application via airbrush gun. Photographs obtained with a transmission light microscope.

8. Sequencing of Test Peptides by MALDI-TOF MS/MS (fragmentation)

For MS/MS experiments, beads of the four test peptides (DYKDDDDKGGTERSGG, DTHFPIGGGGTERSGG, VPFHTDGGGGTERSGG, KFVPFKKSGGTERSGG) were immobilized on a chip, peptides were cleaved from the beads and matrix was applied as previously described. Sequencing was performed using a Bruker autoflex II smartbeam (*Bruker Daltonics* GmbH, Leipzig, Germany) with a MALDI laser source (355 nm, 200 Hz) and a TOF detector. MS spectra were gained in positive reflectron mode with an average of 500 laser shots directly on the resin bead. The precursor ions from three beads of each peptide were selected by taking the most intense peaks, corresponding to the theoretical mass of peptides. Fragmentation spectra were recorded using on average 5500 laser shots, using a precursor mass window of m/z = ± 10. Argon was used as collision gas. Data processing and database search were performed by using a custom-made peptide library search tool (Seq2SpecPeptides), automatically identifying the corresponding peptide sequences. Ion scores were introduced to rank the goodness of identification. The software is based on a code [2] used for the sequence identification of PEG-block-Tla/Michael-oligomers [3]. The results are shown in Tables S1–S4.

Table S1. The three highest ranked sequences of the test peptide DYKDDDDKGGTERSGG (m/z 1770.8) sorted by score corresponding to matching fragments obtained from three independent beads.

Bead	Detected Parent Ion mass (m/z)	Sequence	Score
		TTPKIYYKGGGTERSGG	174
1	1770.9	KDIYYSPKGGGTERSGG	172
		KDIYHIPKGGGTERSGG	172
		KDTYFDPKGGGTERSGG	162
2	1770.9	KDTYFPDKGGGTERSGG	162
		DKTYFDPKGGGTERSGG	160
		TDKTHHFKGGGTERSGG	138
3	1770.9	TDKTHFHKGGGTERSGG	138
		KPYGHHFKGGGTERSGG	136

Table S2. The three highest ranked sequences of the test peptide VPFHTDGGGGTERSGG (m/z 1586.7) sorted by score corresponding to matching fragments obtained from three independent beads.

Bead	Detected Parent Ion mass (m/z)	Sequence	Score
		SVGHTDDVGGGTERSGG	160
1	1586.8	SVGHTDVDGGGTERSGG	160
		SVGHTTIDGGGTERSGG	160
		SGISHDVDGGGTERSGG	150
2	1586.8	SGTVHDVDGGGTERSGG	150
		SGISHTIDGGGTERSGG	150
		SGISHDTIGGGTERSGG	158
3	1586.8	SGTVHDTIGGGTERSGG	158
		SGISHTDIGGGTERSGG	158

Table S3. The three highest ranked sequences of the test peptide KFVPFKKSGGTERSGG (m/z 1737.9) sorted by score corresponding to matching fragments obtained from three independent beads.

Bead	Detected Parent Ion mass (m/z)	Sequence	Score
		KKTDYKVVGGGTERSGG	118
1	1737.9	KKTYDKVVGGGTERSGG	118
		KKSKKFKSGGGTERSGG	110
		VYPIYKVVGGGTERSGG	154
2	1737.9	VYPIFKDVGGGTERSGG	154
		KKSKFKKSGGGTERSGG	152
		HTGGFPDIGGGTERSGG	132
3	1737.9	HTSSSPDIGGGTERSGG	130
		DIPSSPDIGGGTERSGG	130

Table S4. The three highest ranked sequences of the test peptide DTHFPIGGGGTERSGG (m/z 1600.8) sorted by score corresponding to matching fragments obtained from three independent beads.

Bead	Detected Parent Ion mass (m/z)	Sequence	Score
		KKSKFKKSGGGTERSGG	154
1	1600.8	KITFFTHSGGGTERSGG	152
		KITFFHTSGGGTERSGG	152
		DIGVDVIIGGGTERSGG	150
2	1600.8	TSPVDVIIGGGTERSGG	150
		DIGVDPDIGGGTERSGG	150
		DTGIIPDIGGGTERSGG	138
3	1600.8	DTHFPGGIGGGTERSGG	136
		IPGTIVFPGGGTERSGG	132

9. Ladder-sequencing of test peptides by MALDI-TOF MS

For the sequence determination by ladder-sequencing, beads of the four test peptides (DYKDDDDKGGTERSGG, DTHFPIGGGGTERSGG, VPFHTDGGGGTERSGG, KFVPFKKSGGTERSGG) were immobilized on a chip, the peptides were cleaved from the beads and matrix was applied as previously described. MS spectra were obtained in positive reflectron mode with an average of 1500 laser shots directly on the resin bead. The obtained ladder-sequence spectra were processed by using a custom-made peptide library search tool (Seq2SpecPeptides), automatically identifying the corresponding peptide sequences. The peptide sequences are sorted by the number of the recognized truncated peptide sequences. The results are shown in Tables S5–S8.

Table S5. The three highest ranked sequences of the test peptide DYKDDDDKGGTERSGG (m/z 1770.8) sorted by truncated peptides (TP) recognized obtained from three independent beads.

Bead	Detected Parent Ion Mass (m/z)	Sequence	TP
		DYKDDDDKGGGTERSGG	9
1	1770.7	DYKDDDKDGGGTERSGG	8
		DKYDDDDKGGGTERSGG	8
		DYKDDDDKGGGTERSGG	9
2	1770.8	DYKDDDKDGGGTERSGG	8
		DKYDDDDKGGGTERSGG	8
		DYKDDDDKGGGTERSGG	9
3	1770.8	DYKDDDKDGGGTERSGG	8
		DKYDDDDKGGGTERSGG	8

Table 6. The three highest ranked sequences of the test peptide VPFHTDGGGGTERSGG (m/z 1586.7) sorted by truncated peptides (TP) recognized obtained from three independent beads.

Bead	Detected Parent Ion Mass (m/z)	Sequence	TP
		VPFHTDGGGGGTERSGG	9
1	1586.8	VPFDTHGGGGGTERSGG	8
		VPFTDHGGGGGTERSGG	8
		VPFHTDGGGGGTERSGG	9
2	1586.9	VPFHDTGGGGGTERSGG	8
		VPFTHDGGGGGTERSGG	8
		VPFHTDGGGGGTERSGG	9
3	1586.6	VPFHDTGGGGGTERSGG	8
		VPFTHDGGGGGTERSGG	8

Table S7. The three highest ranked sequences of the test peptide KFVPFKKSGGTERSGG (m/z 1737.9) sorted by truncated peptides (TP) recognized obtained from three independent beads.

Bead	Detected Parent Ion Mass (m/z)	Sequence	TP
		KFVPFKKSGGGTERSGG	9
1	1737.9	KFVPKFKSGGGTERSGG	8
		KFPVFKKSGGGTERSGG	8
		KFVPFKKSGGGTERSGG	9
2	1738.1	KFVPKFKSGGGTERSGG	8
		KFPVFKKSGGGTERSGG	8
		KFVPFKKSGGGTERSGG	9
3	1738.0	KFVPKFKSGGGTERSGG	8
		KFPVFKKSGGGTERSGG	8

Table S8. The three highest ranked sequences of the test peptide DTHFPIGGGGTERSGG (m/z 1600.8) sorted by truncated peptides (TP) recognized obtained from three independent beads.

Bead	Detected Parent Ion Mass (m/z)	Sequence	TP
		DTHFPIGGGGGTERSGG	9
1	1600.7	DTHPFIGGGGGTERSGG	8
		TDHFPIGGGGGTERSGG	8
		DTHFPIGGGGGTERSGG	9
2	1600.8	DTHPFIGGGGGTERSGG	8
		TDHFPIGGGGGTERSGG	8
		DTHFPIGGGGGTERSGG	9
3	1600.8	DTHPFIGGGGGTERSGG	8
		TDHFPIGGGGGTERSGG	8

10. Incubation with Anti-FLAG Antibody M2

The chip with the immobilized peptide library 1 was pre-swollen in PBS-T BSA (pH 7.5; 0,1 % Tween 20; 1 % BSA). For prescreening, the chip was incubated with anti-Mouse IgG-Atto 633 antibody diluted 1:10,000 in PBS-T BSA for 1 h to identify false-positive beads. After incubation, the slide was removed from the solution, washed three times with lab water and directly transferred to the fluorescence scanner. Fluorescence scanning was performed using a Microarray Scanner MArS (*Ditabis AG*, Pforzheim, Germany) with a laser wavelength of 532 nm to locate the beads by autofluorescence of the beads and 635 nm for detecting bound, labeled antibody. The PMT gain was set to 65% for 532 nm and 70% for 635 nm and the resolution to 10 µm. For regeneration, the chip was gently shaken for 30 min in an aqueous 6 M guanidine hydrochloride solution, 5 min in lab water and washed three times with PBS-T BSA). Incubation with monoclonal Anti-FLAG M2 antibody diluted 1:10,000 in PBS-T BSA for 15 h and washed five times with PBS-T BSA. Washing was followed by incubation with the anti-Mouse IgG-Atto 633 antibody diluted 1:10,000 in PBS-T BSA for 1 h. Fluorescence scanning was performed as previously described. 33 beads were selected for peptide sequencing, which showed at least three times the fluorescence intensity compared to the prescreening scan (Figure S3).

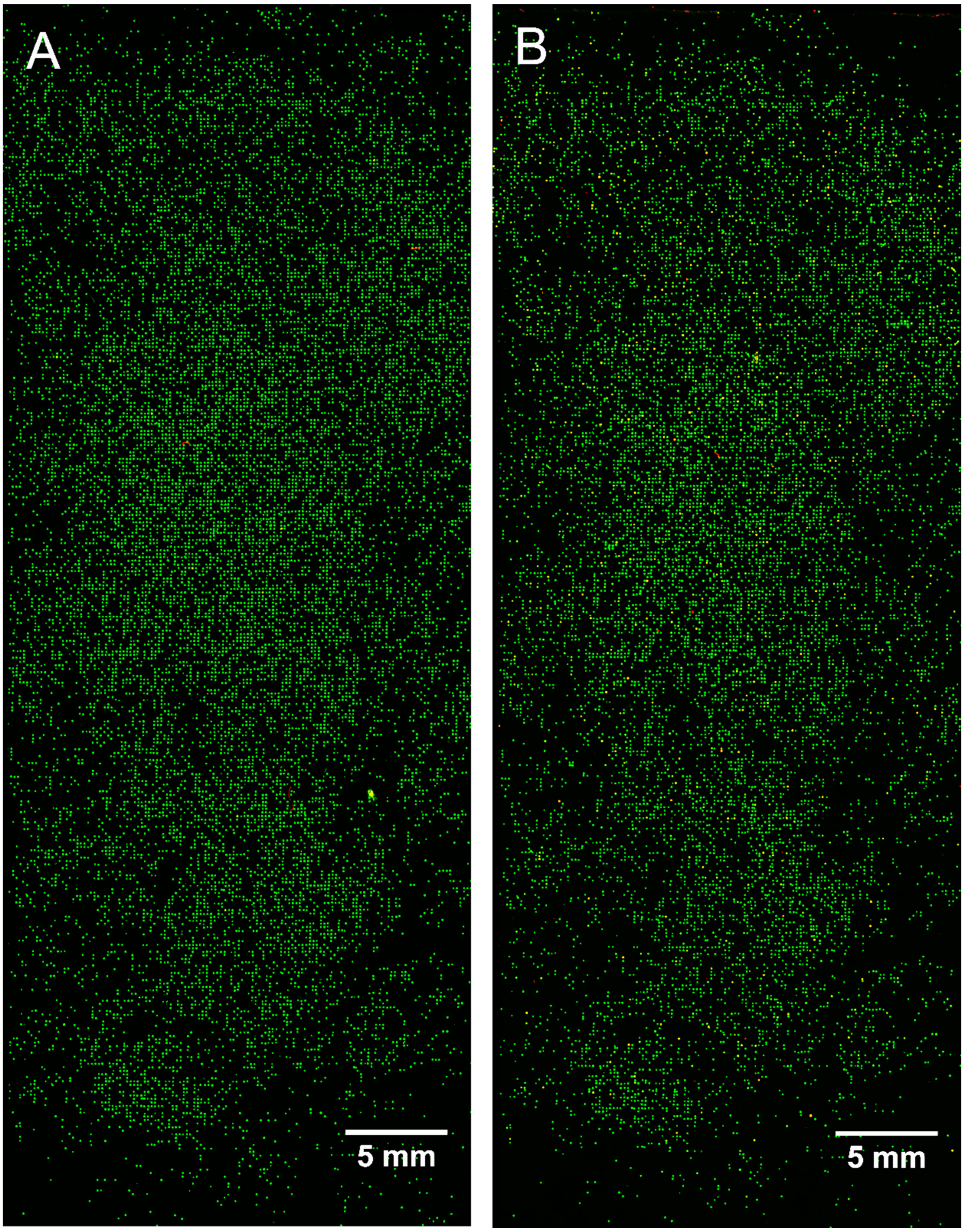

Figure S3. fluorescence scan (10×10 µm per pixel) of a chip with around 12,000 immobilized beads of peptide library 1 (**A**): Prescan after incubation with staining antibody anti-mouse IgG Atto633. (**B**): Scan after incubation with anti-FLAG antibody and staining with anti-mouse IgG Atto633. Overlay of green (excitation 532 nm, emission 570 nm) and red (excitation 635 nm, emission 670 nm) fluorescence. Green fluorescence was used to locate all TentaGel beads by autofluorescence and red fluorescence was used to identify beads bound by the monoclonal anti-FLAG antibody M2.

11. Ladder Sequencing of the Peptides Selected from Library 1 after Anti-FLAG Antibody Incubation

Coordinates obtained from the incubation experiments were transformed into coordinates compatible with the Bruker software. This was achieved by selecting four beads on the corners of the chip in the fluorescence scan and correlating the coordinates by linear regression in an excel sheet in the MALDI software. MS spectra were gained in positive reflectron mode with an average of 1500

laser shots directly on the resin bead. The obtained ladder sequence spectra were processed by using a custom-made peptide library search tool (Seq2Spec, see *ESI*), automatically identifying the corresponding peptide sequences. The peptide sequences are shown in Table S9.

Table S9. Sequences of peptides bound by anti-FLAG antibody M2.

Bead	Pos 1	Pos 2	Pos 3	Pos 4	Pos 5	Pos 6	Pos 7	Pos 8
1	D	Y	K	D	T	D	I	D
2	Y	D	D	Y	K	D	Y	D
3	D	D	Y	K	T	K	D	Y
4	T	Y	K	D	I	D	Y	Y
5	D	Y	I	D	Y	D	Y	K
6	Y	D	D	Y	K	T	D	D
7	Y	Y	D	I	D	I	T	Y
8	Y	Y	K	D	Y	D	T	D
9	Y	Y	D	Y	Y	I	I	D
10	D	Y	K	D	Y	K	D	T
11	I	Y	I	D	Y	K	D	I
12	Y	K	D	T	D	T	T	Y
13	D	Y	K	K	Y	D	I	I
14	D	Y	K	D	Y	Y	Y	D
15	I	Y	D	I	I	T	T	I
16	Y	K	Y	D	D	I	K	T
17	D	Y	D	I	Y	I	Y	D
18	D	Y	K	Y	D	D	Y	D
19	D	Y	D	I	I	Y	I	D
20	Y	Y	D	Y	I	I	I	D
21	D	Y	K	Y	Y	D	Y	T
22	D	Y	D	I	Y	I	D	T
23	D	Y	K	D	Y	D	K	D
24	Y	T	D	Y	K	Y	I	D
25	Y	Y	D	I	Y	I	T	D
26	Y	Y	D	I	I	I	Y	D
27	I	D	Y	K	D	T	D	Y
28	Y	Y	D	I	D	I	I	T
29	Y	Y	D	I	D	I	Y	I
30	Y	Y	D	I	I	Y	D	T
31	I	Y	D	Y	I	Y	T	D
32	Y	Y	D	Y	Y	T	I	D
33	D	Y	D	Y	I	T	I	T

Notes: Color code: Blue = basic AA, red = acidic AA, yellow = hydrophobic AA, orange = aromatic AA, green = polar AA.

12. Ladder Sequencing of the Peptides Selected from Library 2 after Polyclonal Mouse IgG Incubation

The chip with the immobilized peptide library 2 was pre-swollen in PBS-T BSA. Prescreening was performed with the anti-Mouse IgG-Atto 633 antibody as previously described. After regeneration, the chip was incubated with polyclonal mouse IgG diluted 1:10,000 in PBS-T BSA for 15 h, washed five times with PBS-T BSA and fluorescence of the beads was detected as in the anti-FLAG incubation. Fluorescence scanning was performed as previously described. Nine beads were selected for peptide sequencing, which showed at least a three times higher fluorescence intensity compared to the prescreening (Figure S4).

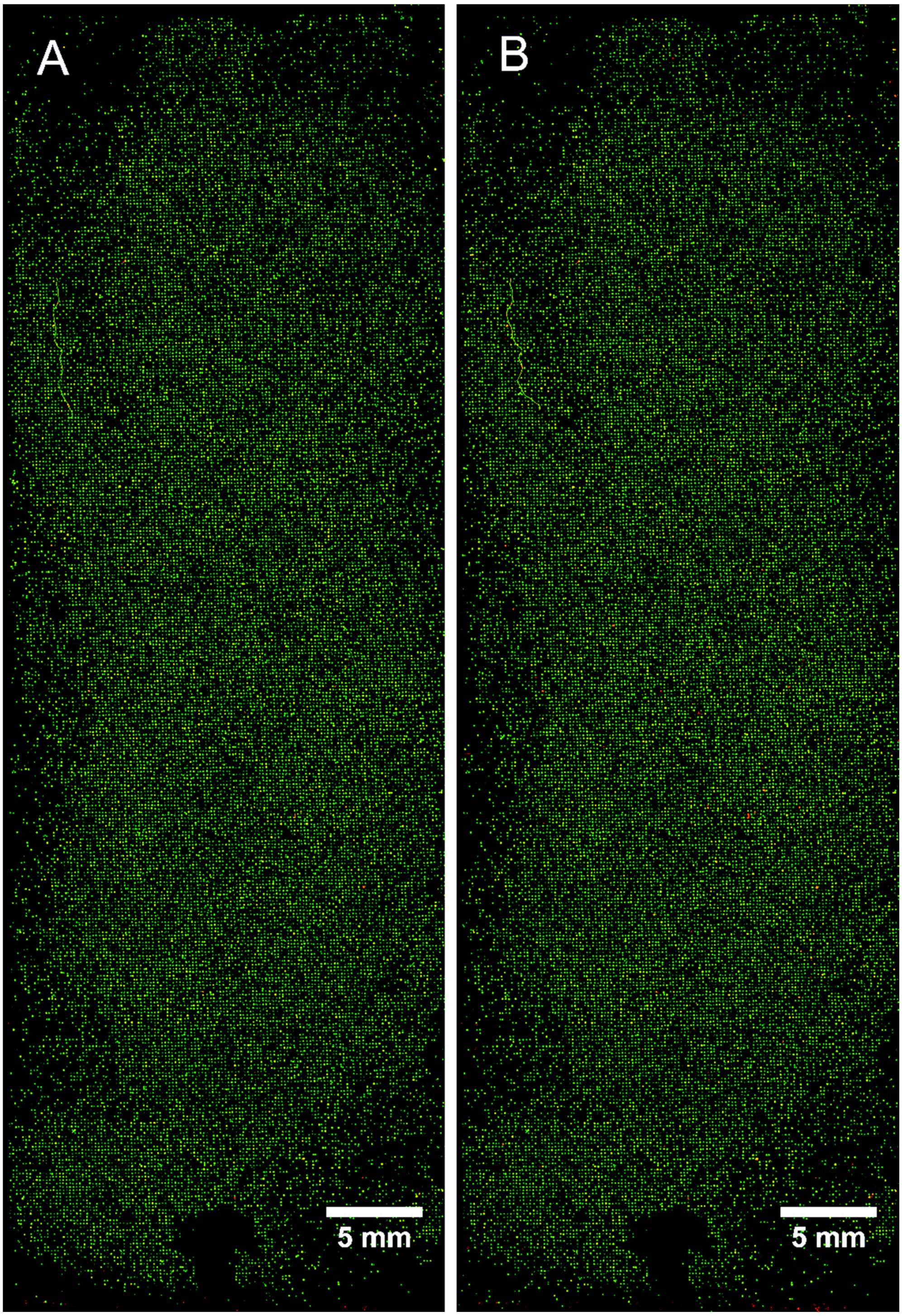

Figure S4. fluorescence scan (10×10 µm per pixel) of a chip with around 12,000 immobilized beads of peptide library 2 (**A**): Prescan after incubation with staining antibody anti-mouse IgG Atto633. (**B**): Scan after incubation with mouse IgG and staining with anti-mouse IgG Atto633. Overlay of green (excitation 532 nm, emission 570 nm) and red (excitation 635 nm, emission 670 nm) fluorescence. Green autofluorescence is used to locate all TentaGel beads and red fluorescence was used to identify beads bound by the polyclonal mouse IgG.

13. Peptide Ladder-sequencing by MALDI TOF MS/MS of Library 2

Coordinates were transformed, and binding peptides were sequenced as previously described.

Table S10. Sequences of peptides bound by polyclonal mouse IgG.

Bead	Pos 1	Pos 2	Pos 3	Pos 4	Pos 5	Pos 6	Pos 7
1	G	R	I	H	G	P	R
2	G	I	H	P	F	G	R
3	I	I	I	F	H	G	R
5	F	G	S	I	I	G	F
6	I	F	F	P	P	H	R
8	F	F	S	P	S	P	R
9	F	R	H	F	S	F	R
10	I	E	E	E	P	G	I
11	G	I	R	F	H	F	F
12	R	H	R	H	R	F	F
13	H	I	G	R	G	R	R
14	G	E	P	F	S	I	P
15	E	H	G	E	P	I	P
18	S	H	S	G	I	F	R

Color code: Blue = basic AA, red = acidic AA, yellow = hydrophobic AA, orange = aromatic AA, green = polar AA.

14. SPR Chip Preparation

Surface Plasmon Resonance (SPR) experiments were performed by a Reichert SR7500DC dual channel SPR system (Reichert, USA). As a flow buffer, HBS-EP (0.01 M HEPES pH 7.4, 0.15 M NaCl, 3 mM EDTA, 0.005% Tween 20) was used throughout all experiments. Immobilization of the antibody was performed on a gold biosensor chip HC200 (XanTec bioanalytics, Germany) using the following amine coupling protocol: Coupling buffer was prepared by dissolving EDC (0.1 M) in MES/NHS buffer (50 mM MES pH 6.0, 0.1 M NHS) and directly injected over both the sample and reference channel for 4 min (20 µl/min). 10 µg of the antibody was diluted in 100 µl of 1 mM sodium acetate (pH 5.0) and injected over the sample channel for 1.5 min (50 µl/min). For quenching, 1 M ethanolamine in lab water was injected over both, the sample and reference channel for 1 min (20 µl/min).

15. SPR Characterization of Anti-flag Binding Peptides

Two peptides identified in the anti-FLAG antibody screening, the original FLAG-peptide and a peptide created as a consensus peptide by choosing the most abundant amino acid at every position, were synthesized by *peptides & elephants GmbH* (Hennigsdorf, Germany). For the determination of their affinity, an SPR chip was prepared as previously described using the monoclonal anti-FLAG antibody, clone M2. 1 µM of the peptides was dissolved in 1 mL HBS-EP buffer. The stock solution was diluted resulting in the following concentrations: 6 nM, 18 nM, 55 nM, 166 nM, 500 nM and 1000 nM. The peptides affinities were measured by injecting the dilutions over both the sample and reference sides for 3.5 min (20 µl/min). The signal measured on the reference side was subtracted from the signal measured on the sample side. For determination of the affinity constant, the signal intensity of the equilibriums state was plotted against the concentration of peptide injected. The calculation was done by the TraceDrawer Data Analysis Software. The results are shown in Figure S5.

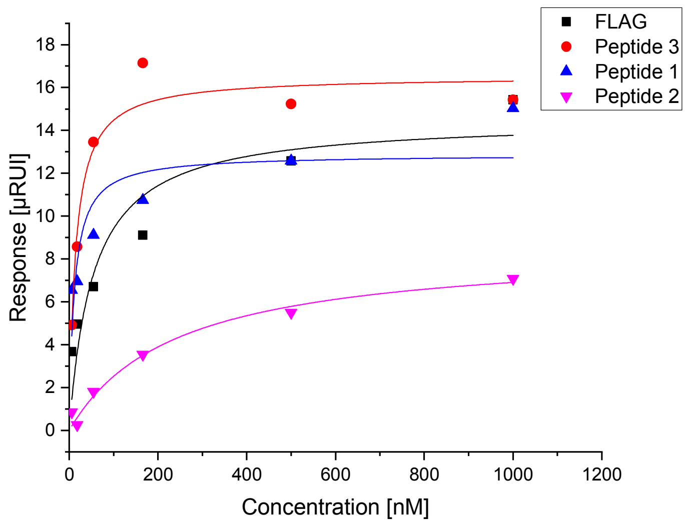

Figure S5. binding results for FLAG (DYKDYDKD), peptide 1 (DYKDYDKD) a FLAG epitope-containing sequence, peptide 2 (DYIDYDYK) a mimotope-containing sequence and peptide 3 (DYDIYIYD) representing a consensus sequence of the most abundant amino acids at each position. The FLAG antibody was immobilized on an HC200 chip and peptide solutions in different concentration were injected. The intensity of the signal in equilibrium is plotted against the concentration.

16. SPR Characterization of Mouse IgG Binding Peptides

Four peptides identified in the mouse IgG screening were resynthesized using standard Fmoc peptide synthesis. For the determination of the affinity, an SPR chip was prepared as previously described using the mouse IgG. 10 µM of the respective peptide was dissolved in 1 mL HBS-EP buffer. The stock solution was diluted, resulting in the following concentrations: 1 µM, 5 µM, 10 µM, 50 µM, 100 µM. The peptide affinities were measured by injecting the dilutions over both the sample and reference sides for 5 min (10 µl/min). For the determination of the affinity constant, the signal intensity of the equilibrium state was plotted against the concentration of peptide injected. The calculation was done by the TraceDrawer Data Analysis Software. The results are shown in Figure S6.

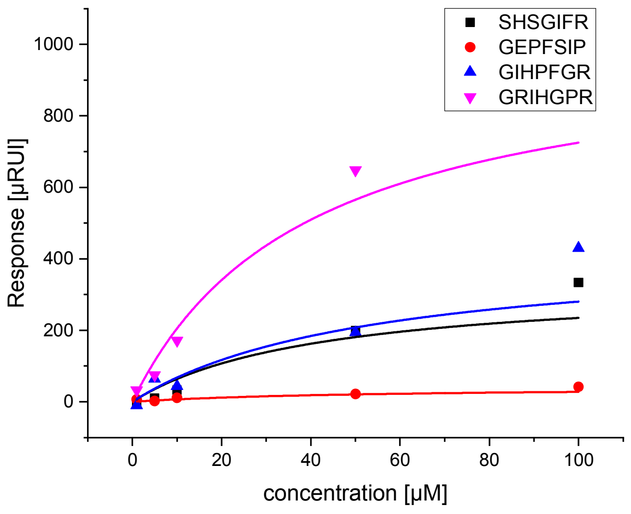

Figure S6. binding results for mouse IgG binding peptides. Mouse IgG was immobilized on an HC200 gold chip and peptide solutions in different concentration were measured. The intensity of the signal in equilibrium is plotted against the concentration.

17. Monitoring of Dibenzofulvene-Piperidine Adducts in Ladder Synthesis

The Fmoc-protecting group was cleaved off by 20% piperidine in DMF for 30 min. Fmoc-screening of the deprotection solution was performed by recording the specific absorption of the dibenzofulvene-piperidine adduct at 300 nm. Dibenzofulvene-piperidine adduct monitoring of two test peptides (DTHFPI-Linker, VPFHTD-Linker) are shown in Figure S7.

Due to inconsistent dibenzofulvene-piperidine adduct formation in the split and mix procedure, a Fmoc-adduct screening during the library synthesis was not performed.

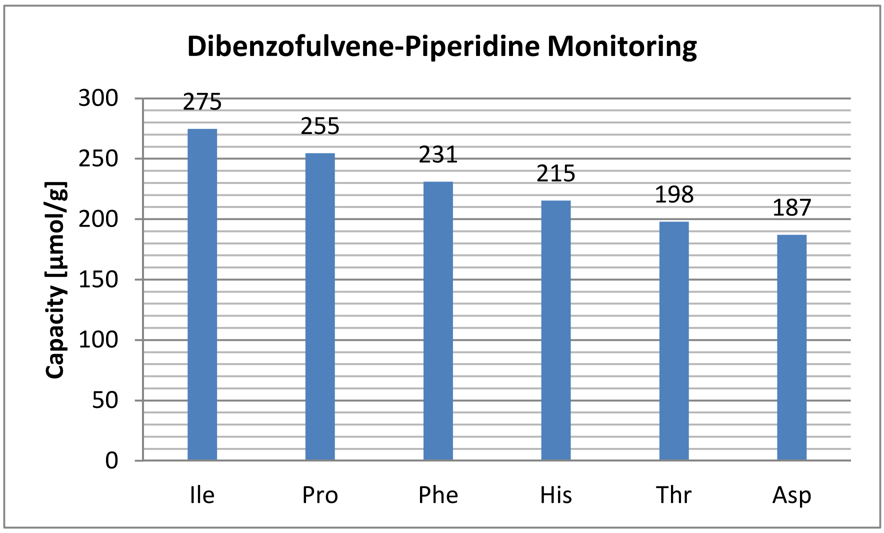

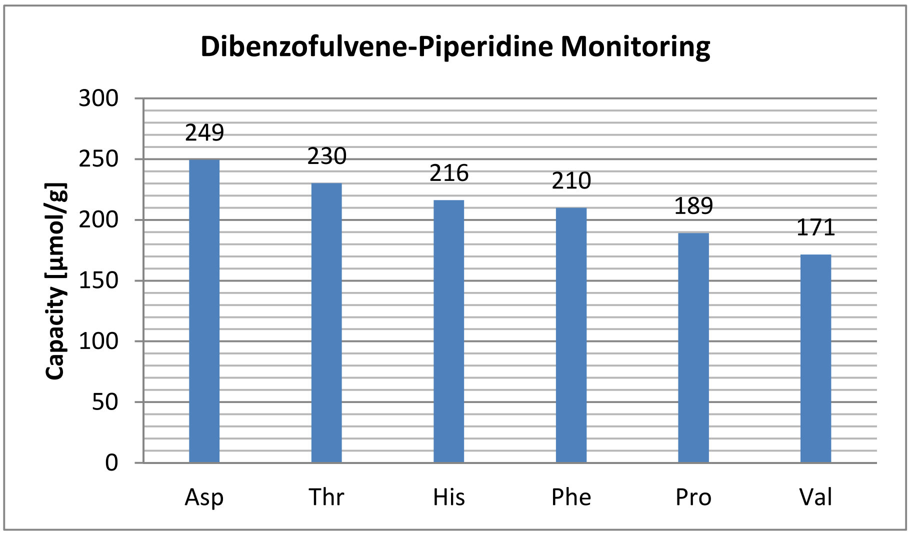

Figure S7. Adduct monitoring of the test peptides DTHFPI and VPFHTD, both linker sequences.

References

1. Chan, P.W.W. Fmoc Solid Phase Peptide Synthesis, Oxford University Press, Oxford, UK, 2000.
2. Schwaar, T.; Remmler, D.; Börner, H.G. Software Spec2Seq. Available online: https://edoc.hu-berlin.de/handle/18452/19953, Humboldt-Universität zu Berlin, 2018 (Accessed on 18 April 2019).
3. Celasun, S.; Remmler, D.; Schwaar, T.; Weller, M.G.; Du Prez, F.; Börner. H.G. Digging into the Sequential Space of Thiolactone Precision Polymers: A Combinatorial Strategy to Identify Functional Domains. *Angew. Chem. Int. Ed.* **2019**, *58*, 1960–1964.

© 2019 by the authors. Submitted for possible open access publication under the terms and conditions of the Creative Commons Attribution (CC BY) license (http://creativecommons.org/licenses/by/4.0/).
